# The development of the evidence-based SDM^MCC^ intervention to improve shared decision making in geriatric outpatients: the DICO study

**DOI:** 10.1186/s12911-020-1022-6

**Published:** 2020-02-19

**Authors:** Ruth E. Pel-Littel, Julia C. M. van Weert, Mirella M. Minkman, Wilma J. M. Scholte op Reimer, Marjolein H. van de Pol, Bianca M. Buurman

**Affiliations:** 1 Department of Internal Medicine, Section of Geriatric Medicine, Amsterdam UMC, University of Amsterdam, Amsterdam, the Netherlands; 2 Vilans, National Center of Expertise for Long-term Care, Vilans, PO Box 8228, 3503 RE Utrecht, The Netherlands; 3 Amsterdam School of Communication Research, University of Amsterdam, Amsterdam, the Netherlands; 4 TIAS School for Business and Society, Tilburg University, Tilburg, the Netherlands; 5 ACHIEVE, Center of Expertise, Faculty of Health, Amsterdam University of Applied Sciences, Amsterdam, the Netherlands; 6 Department of Cardiology, Amsterdam UMC, University of Amsterdam, Amsterdam, the Netherlands; 7 Department of Primary and Community Care, Radboud University Medical Center Nijmegen, Nijmegen, the Netherlands

**Keywords:** Older adults, Multiple chronic conditions, Co-creation, Geriatricians, Training, Preparatory tool

## Abstract

**Background:**

Shared decision making (SDM) contributes to personalized decisions that fit the personal preferences of patients when choosing a treatment for a condition. However, older adults frequently face multiple chronic conditions (MCC). Therefore, implementing SDM requires special features. The aim of this paper is to describe the development of an intervention to improve SDM in older adults with MCC.

**Methods:**

Following the Medical Research Council framework for developing complex interventions, the SDM^MCC^ intervention was developed step-wise. Based on a literature review and empirical research in a co-creation process with end users, we developed training for geriatricians and a preparatory tool for older patients with MCC and informal caregivers. After assessing feasibility, the intervention was implemented in a pilot study (*N* = 108) in two outpatient geriatric clinics of an academic and a non-academic teaching hospital in Amsterdam, the Netherlands.

**Results:**

Key elements of the training for geriatricians include developing skills to involve older adults with MCC and informal caregivers in SDM and following the six-step ‘Dynamic model for SDM with frail older patients’, as well as learning how to explore personal goals related to quality of life and how to form a partnership with the patient and the informal caregiver. Key elements of the preparatory tool for patients include an explicit invitation to participate in SDM, nomination that the patient’s own knowledge is valuable, invitation to form a partnership with the geriatrician, encouragement to share information about daily and social functioning and exploration of possible goals. Furthermore, the invitation of informal caregivers to share their concerns was also a key element.

**Conclusions:**

Through a process of co-creation, both training for geriatricians and a preparatory tool for older adults and their informal caregivers were developed, tailored to the needs of the end users and based on the ‘Dynamic model of SDM with frail older patients’.

## Background

Shared decision making (SDM) reaches out towards decisions about treatment and care that are tailored to a patient’s personal preferences [1]. The benefits of SDM among older adults are a better understanding of harms and benefits, risk perception and less decisional conflict [2, 3]. However, SDM in older adults with MCC, faces several barriers. Decisions about treatment choices for one disease are more difficult, as the co-existing conditions have to be taken into account [4]. The best treatment for a single disease might not be the same as the best treatment for a patient with MCC. Instead of disease-specific outcomes, for many older adults with MCC, maintaining (functional) independence, decreasing specific symptoms or functional challenges (such as being able to walk the dog) and quality of life are considered important goals and priorities [5]. This requires an approach of SDM in which, prior to discussing the benefits and harms of treatment options, personal goals are explored. Furthermore, older adults vary in whether they want to and can participate in SDM, and this also depends on the type of decision that has to be made [6, 7]. Factors that influence the low participation of older adults with MCC in SDM include a perceived lack of knowledge, low self-efficacy, fear, cognitive decline and the belief that there are no options (Pel-Littel RE, Snaterse M, Teppich NM, Buurman BM, van Etten van F, van Weert van JCM, et al. Barriers and facilitators of shared decision making in older patients with chronic conditions; a systematic review. submitted). Older adults with MCC who want to participate in SDM need to be empowered to participate in this process. Moreover, SDM with older people with MCC is often characterized by ‘triadic decision making’, which refers to a decision-making process in which three parties are involved: the health professionals, an adult patient and an adult companion (informal caregiver) [8]. Informal caregivers, such as family members or friends who have caring relationships with the older patients often play an important role in SDM, either because they represent the patient by providing information or because they have their own interests in the decision due to extensive frailty, caring feelings and increasing dependence of their relative [9–13].

**Fig. 1:**
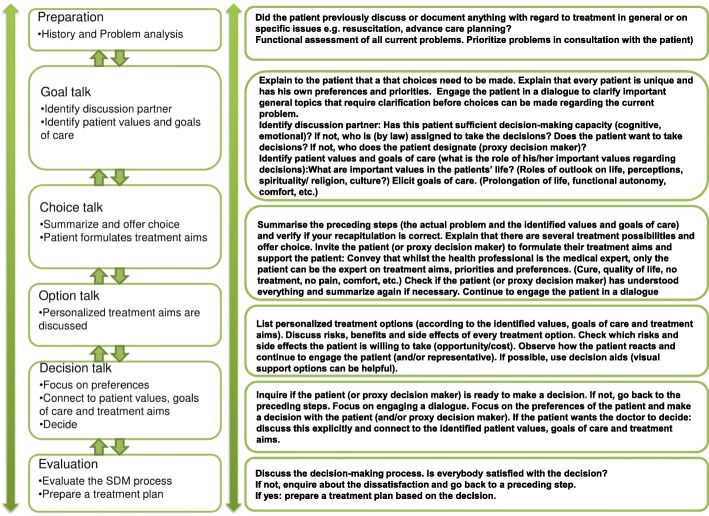
Dynamic model for SDM in frail older patients [15]

To address these needs, van der Pol et al. (2016) developed the ‘Dynamic model of SDM with frail older patients’ [14]. This model builds on existing models for SDM in the general population but adds several dynamic steps to address important issues for SDM with older patients with MCC, such as discussing personal goals related to quality of life and MCC, discussing roles in decision making and evaluating the decision process. A schematic version of this model is depicted in Fig. 1. To bridge the gap between a theoretic model and the daily practice we should explore what is needed for both health professionals as well as older adults to implement the principles of SDM for older adults with MCC in healthcare conversations.

The aim of this research is to develop, pilot test and implement an intervention, called SDM^MCC^, with the primary objective of an increased level of SDM for geriatricians, older adults with MCC and their informal caregivers visiting geriatric outpatient clinics. To this aim, we will explore what is needed for geriatricians, older adults with MCC and informal caregivers to implement the ‘Dynamic model of SDM with frail older patients’ at geriatric outpatient clinics. As part of this aim, we will also examine whether and how existing patient tools could be used to help older adults with MCC and their informal caregivers prepare for a consultation at the geriatric outpatient clinic.

## Methods

**Fig. 2:**
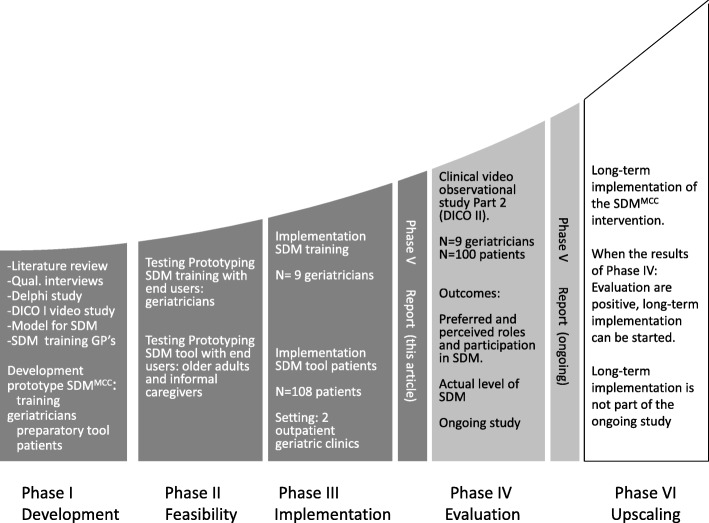
The development of the SDM^MCC^ intervention based on the Medical Research Council framework [16]. *The dark grey parts of this figure have been finished and are described in this article. The light grey column refers to an ongoing evaluation study*

To develop the SDM^MCC^ intervention, the Medical Research Council (MRC) framework was used [16]. This framework provides guidance on the development, pilot testing, implementation and evaluation of complex health interventions [16]. The phases of the MRC framework are (I) development of an intervention, (II) studying feasibility/pilot testing, (III) implementation, (IV) evaluation, (V) reporting and (VI) upscaling of the intervention. In this article, we report (V) about phases I - III (Fig. 2).

### Phase I | development

The aim of the development phase is to identify a coherent theoretical basis guiding the systematic development of the SDM^MCC^ intervention. For this, we conducted a systematic literature review of barriers and facilitators to SDM as experienced by health professionals, older adults with MCC and their informal caregivers. This was expanded with empirical research by means of a qualitative content analysis of structured interviews, a Delphi study and the DICO I study: Decision making In Complex Old populations). This was a video observational study with cross-sectional assessment of interaction during (usual care) medical consultations between geriatricians (*n* = 10), patients (*n* = 108) and informal caregivers (*n* = 68) by three calibrated raters at the geriatric outpatient department of two Dutch hospitals (after the implementation of the SDM^MCC^ intervention we are currently conducting the DICO II study with a similar design and a comparable patient group to study the effect of the SDM^MCC^ intervention). The results of these studies are summarized in this article and reported in detail elsewhere [17–19]. The analysis of the results guided the development of the first prototype of the SDM^MCC^ intervention, which consisted of SDM^MCC^ training for geriatricians and a preparatory tool for older adults and their informal caregivers, based on the principles of the ‘Dynamic model of SDM with frail older patients’.

### Phase II | feasibility/pilot testing

In this phase, the prototypes of the SDM^MCC^ training for geriatricians and the preparatory tool for older adults and informal caregivers were pilot tested with end users: geriatricians (*n* = 11), older adults and their informal caregivers (*n* = 10).

The concept of training for geriatricians was discussed in two rounds of semi-structured interviews with geriatricians (*n* = 11). All interview participants were recruited from the professional network of the principal investigator (RPL) and were based in Utrecht, Amsterdam and Hilversum (the Netherlands). The participants had not been previously involved in SDM implementation activities. The inclusion criteria for these geriatricians were that 1) they worked with patients with MCC on a daily basis and 2) geriatrics or internal medicine was their main specialization. After each interview, the concept training was adapted based on the results of the interviews. The semi-structured interviews were recorded and transcribed verbatim afterwards.

The preparatory tool was discussed in three interview rounds. In round 1, older adults (*n* = 3) and informal caregivers (*n* = 2) participated who had visited a geriatric outpatient clinic in the past month. In round 2, older adults (*n* = 5) participated from the professional network of the principal investigator (RPL), who had experience both as a patient and as an informal caregiver, and geriatricians (*n* = 2) from an academic hospital (AMC). In round 3, only the two geriatricians from the AMC participated. The purpose of the tool was explained by the researcher, and participants were invited to react to relevance, user-friendliness and understandability. Additionally, they were asked to react to the pictures that were meant to support the text. After each round, adaptations were made based on the results of the feasibility tests. This resulted in the final SDM^MCC^ training and preparatory tool.

### Phase III | implementation

The training was given to the nine geriatricians of the geriatric outpatient departments of two Dutch hospitals: 1) the Academic Medical Center (AMC) (*n* = 4) and 2) the Medical Center Slotervaart, a non-academic teaching hospital (MC SLV) (*n* = 5). These geriatricians met the following criteria: (1) specialized in geriatrics, (2) working at the outpatient clinic of the geriatric department. Temporary staff was not eligible. The purpose of the training was explained in a staff meeting at each hospital and each geriatrician got a formal invitation to attend the training. At the end of the training session, all participants were asked to evaluate the training by answering four questions: (What are you going to do differently tomorrow? What are your learning points? What grade do you give the training? Do you have any tips or comments?).

Eligible patients who were scheduled for visits at the geriatric outpatient clinics of these hospitals between September 2017 and June 2018 were approached by telephone and informed about the study. If they were willing to receive information about the study, an information letter and the preparatory tool was sent by mail. In the information letter about the study, the purpose of the preparatory tool was explained and patients were requested to fill in the preparatory tool, if possible with their informal caregiver and bring it to the consultation. Of all eligible patients (*n* = 514), 108 consented to participate in the study (21% of all scheduled patients) (see Additional file 1: Flowchart of inclusion). To be eligible for the study patients had to meet the following criteria: 1) sufficient mastery of the Dutch language, 2) a life expectancy of more than 3 months, 3) not having a severe stage of dementia (MMSE < 15) according to the medical file, 4) being a geriatric patient visiting the geriatric outpatient department. A geriatric patient is not merely defined by age, but by decreased functional reserves leading to frailty and the prevalence of more than one chronic condition [20]. In the Netherlands older adults are usually referred to a geriatrician by their General Practitioner when there are MCC often combined with various geriatric syndromes such as falls, cognitive impairment and functional decline. In an information letter about the study, the purpose of the preparatory tool was explained and patients were requested to fill in the preparatory tool, if possible with their informal caregiver and bring it to the consultation. Immediately after the consultation with the geriatrician, the patients were asked if they had received the tool, if they had completed it and what their opinion was about the tool. Furthermore, they were asked if they had discussed the tool with someone else, for example, a family member and if the researchers could receive the completed tool or take a picture of it. Additionally, informal caregivers were asked if they had read the tool, if they had completed the part for informal caregivers and how they appreciated the tool. Written informed consent was obtained from all older adults and informal caregivers.

#### Patient involvement

Both patients and their informal caregivers were involved in the development and testing of the intervention (Additional file 2: GRIPP2 reporting checklist).

## Results

**Fig. 3:**
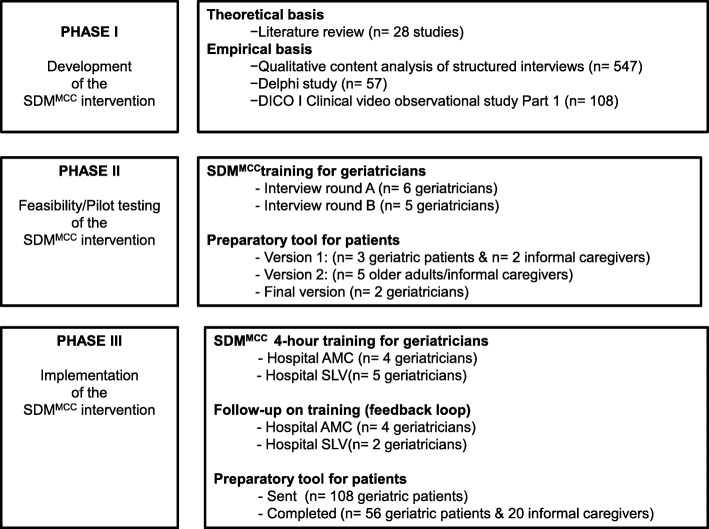
Flowchart of participants in different phases of the study

The participant included in the different phases of the study are presented in a flowchart (Fig. 3).

### Phase I | development

#### Insights from existing literature and empirical evidence

Table 1 depicts the analysis of the systematic literature review of barriers and facilitators to SDM, the qualitative content analysis of structured interviews, the Delphi study and the DICO I video observational study, all used for the development of the intervention. The results of Phase I guided the development of the SDM^MCC^ intervention.

**Table 1:** Results of Phase I

Phase I: Development			
Ia Identifying existing evidence			
*Literature review (n = 28 reviewed studies)*			
Step	Aim	Results	Intervention
			A: for training
			B: for preparatory tool
1	To identify barriers and facilitators to SDM with older adults with MCC	Main barriers:	
		1. Personal patient characteristics such as being in poor health and/or having cognitive or physical impairments.	1. Tailor information to capacity of patient, discuss decision capacity/needs (A)
		2. Feeling no permission to participate in SDM	2. Explicit invitation to participate in SDM: The doctor has medical knowledge - you have knowledge of your personal situation - together, we can decide what is best for you! (AB)
		3. Health professionals with poor interpersonal skills, organizational barriers as time pressure and high turnover of patients.	3. Good patient preparation before consultation enables moving more quickly to the important points of discussion (A)
		Main facilitators:	
		1. The patient’s experiences of living with chronic health conditions	1. Appraisal of personal experiences of patients (A)
		2. The assistance of informal caregivers in decision support.	2. Involvement of informal caregiver in SDM by recognizing their contribution in care and inquiring about their concerns (AB)
		3. An individualized approach where health professionals seek patient’s preferences.	3. Take personal goals into account by asking them what must happen to improve their quality of life (AB)
Ib Gathering additional empirical evidence			
*Qualitative content analysis of structured interviews (n = 547)*			
	To investigate the personal views on the ageing process communicated by older persons	1. Acceptance of ageing, (further) deterioration and worries about limitations and family were important themes on the ageing process communicated by older persons.	1.1 Facilitation of discussion about ‘what matters to you’ by asking them what must happen to improve their quality of life (AB)
			1.2 Discuss personal goals that contribute to quality of life (A)
*Delphi study (n = 57)*			
	To explore what domains of health are important to discuss with a health professional, according to older adults in the Netherlands	1. The domains older adults gained consensus on were:	1.1 Address in goal talk these topics (AB) 1.2 Explore impact of MCC on daily life (A)
		- functional limitations	
		- emotional wellbeing	
		- social functioning	
		- quality of life	
		- coping with stress	
		- dealing with chronic health conditions and the effects of them on daily life	
*DICO I Clinical video observational study Part 1* (n = 108)			
	1. Which decisions face older adults with MCC and their informal caregivers during geriatric visits to the hospital?	1. Main decisions are	1) Share that there are often more options (A)
		- Additional diagnostics	
		- Medication	
		- Follow-up	
		- Referral to primary care (case manager, GP, physiotherapist, psychologist)	
	2. What is the preferred and perceived role and participation in SDM for older adults with MCC and their informal caregivers?	2. Main roles in decision making were for patients: ‘triadic decision making’ 41%<> 33% (preferred <> perceived), and for informal caregivers: ‘triadic decision making’ 71%<> 55% (preferred <> perceived). Preferred and perceived participation was for patients 6.6<> 5.1, for informal caregivers 7<> 5.	2. Discuss how older adults with MCC and informal caregivers want to be involved in decision making. Involve them according to their preferences. (A)
	3. To what extent are steps of the SDM process operationalized by geriatricians during geriatric consultations [15]?	3 The OPTION^MCC^ score was 42.5 (0–100), items about ‘team talk’ and ‘evaluation’ scored the lowest (resp. 31 & 36.5).	3. Train geriatricians in team talk and evaluation phase in the process of SDM. (A)

#### Development of the prototypes

##### Development of the prototype training for geriatricians

An expert panel was composed that consisted of the developer of the ‘Dynamic model of SDM with frail older patients’ (MvdP), a professor of Health Communication, specializing in older adults (JvW), the researcher/project leader, specializing in SDM and older adults (RPL), and a master student in management, policy analysis and entrepreneurship (ABP). As a basis for the training, they used an initial basic training that was previously developed to train General Practitioners in the ‘Dynamic model of SDM with frail older patients’ [14]. This training for General Practitioners was based on a teaching framework and proposed key competencies for SDM with older adults as composed by the original developer of the model [14]. The insights gained in Phase 1 (see Table 1) were plotted in a so-called ‘heat map’ to obtain insight into which current training components should be maintained, which components should be omitted and which elements were lacking and should be added to the training (Additional file 3). In the next step, the training for General Practitioners was modified according to these recommendations, which led to ‘Prototype SDM^MCC^ training geriatricians 1.0’.

In the development of the training, the principles of the Miller learning pyramid were applied [21]. Within this classification, in the form of a pyramid that was developed to determine the competence level of doctors, four levels of competence are distinguished: 1: knowing (knowledge), 2: knowing how (knowledge can be used), 3: showing how (acting in a simulated environment) and 4: doing (acting in everyday practice). Table 2 depicts how the training was structured:

**Table 2:** Structure and learning objectives of the SDM^MCC^ training (final prototype)

Structure of the SDM^MCC^ training	
Knows	Part 1: knowing (knowledge)
	− What does the geriatrician already know, do and feel about SDM? (background, prejudices, pseudo-participation, how you feel about the subject).
	− Introduction to SDM (general model, complexity of older adults with MCC, legal capacity/cognition/decision capacity, views of life and expectations/goals, role of informal caregivers)
Knows how	Part 2: knowing how (to use knowledge)
	− Introduction steps of ‘Dynamic model of SDM with frail older patients’. (discuss for each step how to handle it, examples, discuss decision aids that patients or geriatricians have used)
	− Introduction of preparatory tool for patients and informal caregivers
	− Discussion of cases (CVA, Parkinson’s, falls)
Shows how	Part 3: showing how (acting in a simulated environment)
	− Role-play is used to apply the learned skills with a professional training actor: introducing and practising own case studies of geriatricians
Does	Part 4: doing (acting in everyday practice)
	− ‘Coaching on the job’: geriatricians got feedback from the trainer on a videotaped consultation of their daily routine practice.
Learning objectives after following the training:	
Knowledge	− The participants have insight into the concept of SDM with older adults with MCC and informal caregivers. The participants have knowledge of the ‘Dynamic model of SDM with frail older patients’.
	− The participants know how to apply this model in different situations and how to involve patients and informal caregivers.
Skills	− The participants gained practical skills to apply the model by practicing it with each other and with a professional training actor
	− The participants apply the model in daily clinical practice
Attitude	− The participants have insight into their own behaviors and attitudes towards this subject.
	− The participants have a positive attitude towards SDM with older adults with MCC and can describe the benefits.

##### Development of the prototype preparatory tool for older adults with MCC and informal caregivers

The patient tools were examined to evaluate how they met the formulated recommendations from Phase I (Table 3). All tools contain valuable components, but none of the tools met all recommendations as formulated in Phase I. Additionally, none of the tools focuses on the role of informal caregivers. The prototype of the preparatory tool for older adults with MCC and informal caregivers was therefore developed as a new tool, although we used some aspects of the existing tools. Furthermore, we included elements we found in the international literature about empowering patients in SDM, such as the underlying principles of Question Prompt Lists and the ‘Ask 3 Questions’ campaign in the U.K. Magic program [2, 25–27].

**Table 3:** Description of existing tools for empowerment of patients

	Description	Valuable components that were used in the preparatory tool	Components not feasible for older population with MCC in geriatric consultations
Toolbox Person-centred care [22]	This toolbox aims at supporting people with chronic diseases in primary care consultations. It addresses agenda setting, discusses goals and actions and follow-up. It consists of a part for general practitioners and a part for patients. In the part for patients, patients are explicitly invited to participate in SDM. It stimulates patients to think about what they want to discuss in the consultation. Topics raised by ‘fellow patients’ are given as an example. It emphasizes the teamwork between patient and health professional during the consultation when discussing different options, pros and cons and personal preferences. The tool also comprises space for a follow-up plan. The tool refers more to self-management and lifestyle changes and seems less suitable when facing medical decisions at the outpatient geriatric clinic.	Agenda setting	Aim at primary care
		Goal setting	
		Invitation to SDM	
		Emphasize teamwork	Focus on disease management and lifestyle changes
www.watertoedoet.nl [23]	This is an extensive website aiming at awareness of what is important for patients and helps thinking about personal goals. It provides patients with a final print that they can bring to the clinical encounter. However, it requires moderate digital health literacy from patients, which is not yet common among all geriatric outpatients.	Goal setting	Requiring digital health literacy
		Thinking about quality of life	
Patient’s Action Communication Card PAC-card [24]	A PAC-card can be used by patients during a clinical encounter as a checklist to help them ask questions about their problem and treatment. It has an informative character and focuses on the problem of that actual moment. It does not address the discussion of personal goals or forming a trustful relationship between patient and health professional. The PAC-Card does not focus on the possibility of different options. It was developed to be used as a paper version.	Encouragement to ask questions	No preparation
		Paper version, easy to use	
Ask 3 Questions (Magic Program) [25]	The Ask 3 Questions campaign, part of the U.K. Magic program, is designed to encourage patients to ask questions and play a more active role in decisions about their treatment and care. This audit tool provides a simple checklist to help services assess how well they are promoting materials that encourage people to Ask 3 Questions:	Encourage the patient to ask questions about options, benefits and risks.	Focus on single diseases
	1. What are my options?		
	2. What are the benefits and possible risks?		
	3. How likely are these risks and benefits?		
Question Prompt Lists [26]	A QPL is an inexpensive communication tool consisting of a structured list of questions designed to encourage information gathering. Patients can use these questions as examples which they can choose to discuss during the consultation. By using a QPL, patients are expected to participate more actively during their consultation, for instance by asking more and broader-ranging questions.	Encourage the patient to think in advance about questions to discuss during the consultation	

**Fig. 4:**
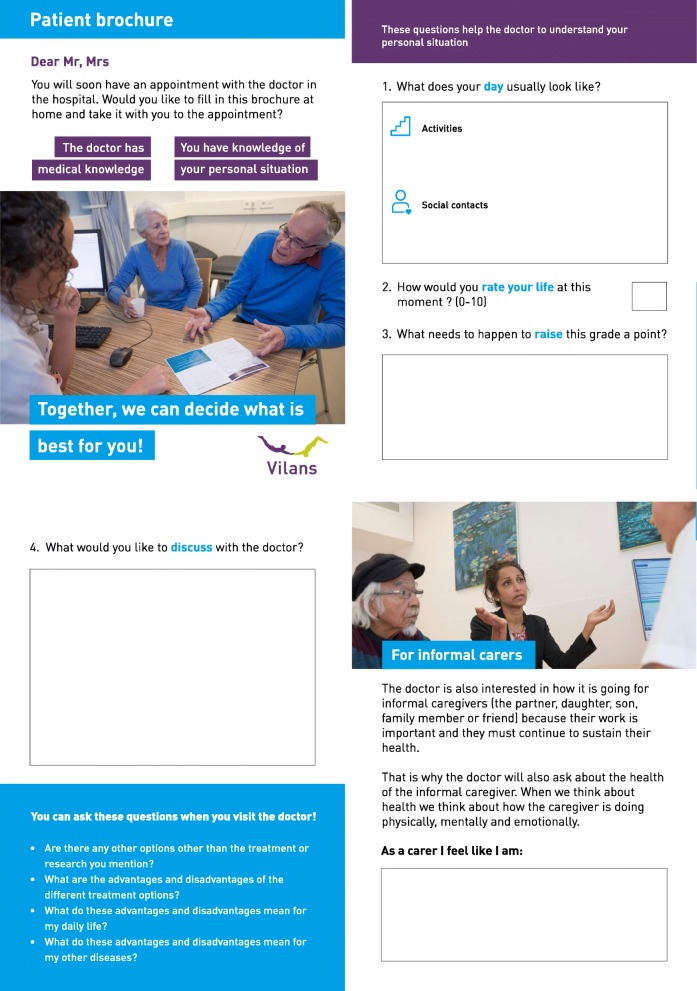
Final preparatory tool for older adults and informal caregivers

The tool (final version is depicted in Fig. 4) consists of 4 pages. Page 1 is an explicit invitation to participate in SDM and an appraisal of the older adult’s personal expertise. Page 2 (1) encourages older adults to describe their daily activities and social contacts (focus on functional limitations and social functioning), (2) asks them to grade their quality of life and (3) inquires about what would be necessary to increase this grade by one point (personal goals). Page 3 helps the older adult prepare for the conversation with the doctor by means of an open question ‘what would you like to discuss with the doctor’ as well as by providing ‘example questions’ that help the older adult inquire about options, harms and benefits and implications for daily life. Page 4 is addressed to the informal caregiver. First, it explains why the geriatrician is also interested in the informal caregiver (recognizing partnership and recognizing the potential burden of informal care). Second, an outcome indicator about informal caregiver burden in dementia care used in the Netherlands was added to the tool [28]. This indicator inquires about how the informal caregiver was feeling in the past month, through indicating their mood according to the rungs of a ladder drawing; furthermore, it asks the informal caregiver to write down three words about the best day in the past month and three words about the worst day in the past month.

### Phase II | feasibility/pilot testing

The aim of this phase was to pilot test the SDM^MCC^ training for geriatricians and the preparatory tool for older adults and informal caregivers and to co-create the final versions with the end users *((*i.e. *geriatricians, geriatric patients and informal caregivers).*

#### Training for geriatricians

In the first interview round (A), the components of the prototype training for geriatricians were presented to six geriatricians. Semi-structured interviews showed that the majority of the participants had a positive attitude towards the training. However, they thought the training should focus more on the problems they encounter in daily practice, such as conflicting visions between geriatricians and patients and how to explore the goals and preferences of patients. These problems were also perceived as most difficult to learn during the training because of the complexity of older people with MCC. Based on these results, the training was adapted to better match daily practice. For example, more practical exercise was organized with a professional training actor. In the second round (B), the adapted training was presented to five other geriatricians. In this round, some components remained the same, but some were added, altered or removed completely. For example, the training focused much more on ‘exploration of goals’, but learning ‘mapping the patients’ history’ was removed, since geriatricians do this already in each new consultation, which might differ from general practitioners. This process led to the final prototype for the training (Table 2).

#### Preparatory tool for older adults and informal caregivers

The first version of the preparatory tool was presented to three older adults with MCC and two informal caregivers. The interviewed older adults thought the first version contained ‘good questions’. However, all the informal caregivers had difficulty with the question addressed to them (the outcome indicator for informal caregivers in dementia care). They did not understand the meaning of ‘ticking a rung of the drawn ladder’ as an indicator about their feeling in the past month and had difficulty writing down three words about the best day in the past month and three words about the worst day in the past month. The informal caregivers proposed a simpler version of the question: ‘As an informal caregiver, I feel........’. Their input was used to develop a second version of the tool. The older adults, informal caregivers and geriatricians in the second round mainly recommended simplifying the language, and the participants gave many suggestions on how to make the text shorter and easier to interpret. They also gave suggestions regarding how to distinguish more clearly between the questions meant for older adults and those for informal caregivers. The drawn pictures in the tool, representing older patients, informal caregivers and geriatricians, were perceived as childish. This feedback guided the third version, and the only comment on the third version was to use a different photo for the front page, which was done in the final version of the preparatory tool.

### Phase III | implementation

#### Implementation of the training for geriatricians

The geriatricians (*n* = 9) of the two outpatient geriatric clinics of the AMC and MC SLV were invited to follow the training. In each hospital, one 4-h training session was organized. In addition, a placemat with the steps of SDM with older adults with MCC was distributed, and the preparatory tool was shown and explained. The training was given by a teacher/researcher/general practitioner specialized in SDM with older adults with MCC from Radboud University in Nijmegen. A professional training actor attended the meeting to provide training opportunities. The principal researcher (RPL) was present to guide the process. In the AMC, two geriatricians followed the 4-h training session, but two other geriatricians were not able to attend that day due to logistic reasons (1) and illness (1). These two geriatricians received an adapted training that consisted of two informative videos made by the trainer, about SDM in general and about the SDM^MCC^ model. This was followed by a meeting with the principal researcher to discuss SDM in older adults with MCC. In the MC SLV, all five geriatricians followed the training. All geriatricians were offered a feedback session with the trainer to discuss the SDM process, using a videotape of one of their daily clinical consultations with the target group. Six geriatricians (4 AMC, 2 MC SLV) were able to participate in this individual feedback session.

The geriatricians who followed the 4-h training session graded the training with an 8 (0–10). They stated that they learned the most about communication strategies and the ‘Dynamic model of SDM with frail older patients’. When asked what they would do differently tomorrow; most of them said ‘Having a real goal talk with my patients.’ They appreciated the safe atmosphere during the training, the handouts and working with the training actor. For following training sessions, they advised using more real-life cases to practice with.

#### Implementation of the preparatory tool for older adults and informal caregivers

As depicted in Table 4, 74 (69%) older adults confirmed that they received the preparatory tool. The tool was filled out by 56 older adults (52%) and 20 (37%) informal caregivers. Of them, 26 older adults (35%) discussed the tool with their informal caregiver. Of the older adults who had filled out the tool, 64% found the tool ‘good, clear or informative’, 8% thought the tool was ‘confusing, difficult’, 7% found the tool ‘limited, too short’, 5% considered the tool not applicable to their situation, 5% had no opinion and 11% had other remarks, mainly about logistics. Of the informal caregivers who had used the tool, 63% rated the tool as ‘good, clear or informative’, 19% considered the tool not applicable to their situation, 7% thought the tool was difficult, 4% found the tool ‘limited, too short’ and 7% had no opinion.

**Table 4:** Implementation of the preparatory tool for older adults and informal caregivers

	Yes (n,%)	
*Patients:* Did you receive the preparatory tool?	74 (68.5)	
*Patients:* Did you complete the preparatory tool?	56 (51.9)	
*Patients:* Did you discuss the preparatory tool with your relatives?	26 (24.1)	
	Patients that used the tool (*n* = 56)	Informal caregivers that used the tool (*n* = 20)
What did you think of the preparatory tool?		
good, clear or informative	64%	63%
confusing, difficult’	8%	7%
limited, too short’	7%	4%
not applicable to their situation	5%	19%
no opinion	5%	7%
other remarks \mainly logistic)	11%	

## Discussion

Our study shows that the SDM^MCC^ implementation intervention has been systematically designed based on both scientific and empirical evidence. The SDM^MCC^ intervention includes SDM training for geriatricians and a preparatory tool for older adults and informal caregivers. Through the process of co-creating with the end users, both products were tailored to the specific needs of older adults and geriatricians.

Key elements of the training for geriatricians in SDM^MCC^ include exploration of the current attitude towards and knowledge and use of SDM among participating geriatricians and a discussion about pseudo-participation and prejudice. This is followed by theory about SDM: general model of SDM, complexity in older adults with MCC, assessing legal capacity of older adults, cognition problems, life expectancy, personal goals, and the role of informal caregivers. The 6-step SDM^MCC^ model should be explained, discussing how to do each step and giving examples. Practical exercise, preferably with real-life cases and a training actor, fosters behavioural change. Key elements of the preparatory tool for older adults include an explicit invitation to participate in SDM, appreciation of older adults’ own knowledge, forming a team, sharing information about daily and social functioning and exploring possible goals. Furthermore, older adults are empowered to prepare what they want to discuss in the encounter, e.g., by example questions for older adults to inquire about options, benefits and harms of each option and potential consequences for other conditions (MCC). Finally, the concerns of informal caregivers are addressed by recognizing partnership and inquiring about the potential burden of informal care. Since the preparatory tool was evaluated positively by more than 60% of both patients and informal caregivers; there seemed to be sufficient ground to proceed with the implementation of the preparatory tool. However, this also shows that there still is room for improvement of the preparatory tool. Therefore, we recommend a continuous evaluation of the use of the preparatory tool in daily practice to generate further improvements, for example through interviewing patients more extensively about the preparatory tool.

To evaluate the SDM^MCC^ intervention, we will evaluate in the DICO II study the effect of SDM training for geriatricians on the level of SDM compared to the level of SDM in the clinical video observation study Part 1 (DICO I) and the effect of the SDM tool for older adults and informal caregivers by comparing the preferred and perceived participation and decision roles and decisional conflict with the findings of the DICO I study.

We expect that the use of the SDM^MCC^ intervention to implement the ‘Dynamic model of SDM with frail older patients’, tailored to the geriatric outpatient setting, will contribute to decisions that comply with personal goals and preferences. Also in recent literature about SDM, we see an increasing awareness of the need to explore personal goals and context in SDM and to support the older adult more through the SDM process [15, 29]. Our findings are in line with Vermunt et al. (2017, 2018), who strongly advocate goal setting as a key element of a person-centred approach when caring for older adults with MCC [5, 30]. The SDM^MCC^ intervention focuses on triadic decision making, following the literature that emphasizes the often important role of informal caregivers of older adults visiting the geriatric outpatient clinic [9–13].

Although the major strength of this research was the co-creation with the end users, i.e., geriatricians as well as older adults and their informal caregivers, the number of those involved was limited. However, proceeding through different rounds, each round presenting an improved version of either the training or the preparatory tool, led, in our opinion, to products that are tailored to the needs of end users.

## Conclusions

This article describes the development, pilot testing and implementation of the evidence-based SDM^MCC^ intervention to improve SDM with older adults suffering from MCC. Through a process of co-creation, both training for geriatricians and a preparatory tool for older adults with MCC and their informal caregivers were developed, tailored to the needs of the end users and based on the ‘Dynamic model of SDM with frail older patients’.

## Supplementary information

**Additional file 1.** Flowchart of the inclusion.

**Additional file 2.** GRIPP2 reporting checklist.

**Additional file 3.** Heatmap Adaptions training SDM for geriatricians.

## Data Availability

The datasets used and/or analyzed during the current study are available from the corresponding author on reasonable request. The preparatory tool can be downloaded for free in English and Dutch at https://www.vilans.org/app/uploads/2019/07/patient-brochure.pdf (English) and https://www.zorgvoorbeter.nl/persoonsgerichte-zorg/samen-beslissen-hulpmiddelen (Dutch).

## Abbreviations

AMC: Amsterdam medical center
DICO: Decision making in complex old populations
GRIPP 2: Guidance for reporting involvement of patients and the public
MC SLV: Medical center Slotervaart
MCC: Multiple chronic conditions
MRC: Medical research council
SDM: Shared decision making
SDM^MCC^: Shared decision making with multiple chronic conditions
