# Supplementary figures and images for: The development of the evidence-based SDMMCC intervention to improve shared decision making in geriatric outpatients: the DICO study
Source: BMC Med Inform Decis Mak. 2020 Feb 19;20:35. doi: 10.1186/s12911-020-1022-6 (PMC7031985; doi:10.1186/s12911-020-1022-6)

## Supplementary S1: Flowchart inclusion Phase III Implementation

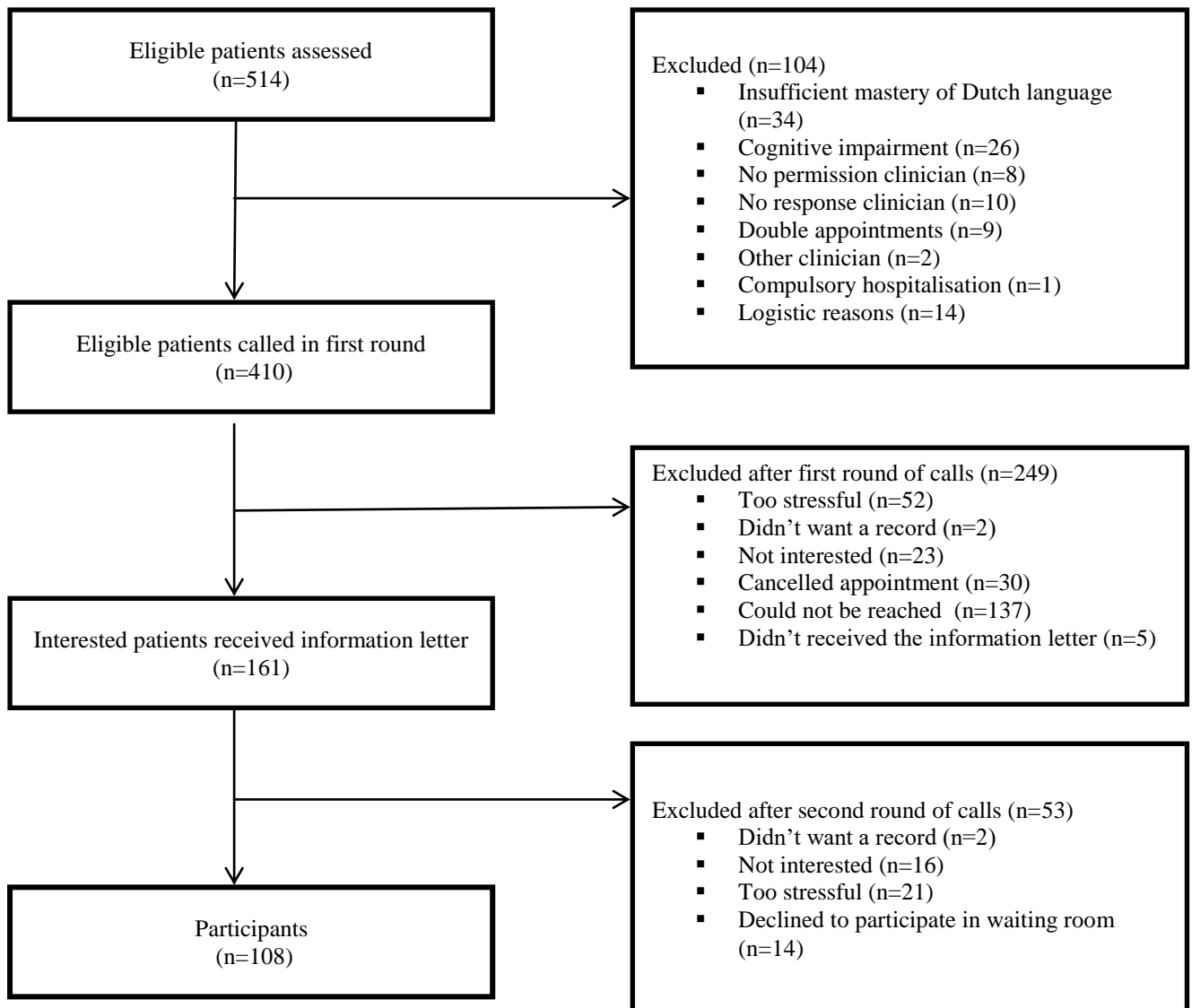

Supplement: Supplementary file 1 — Additional file 1. Flowchart of the inclusion. [file 12911_2020_1022_MOESM1_ESM.pdf]
