# Supplementary material for: The development of the evidence-based SDMMCC intervention to improve shared decision making in geriatric outpatients: the DICO study
Source: BMC Med Inform Decis Mak. 2020 Feb 19;20:35. doi: 10.1186/s12911-020-1022-6 (PMC7031985; doi:10.1186/s12911-020-1022-6)
Supplement: Supplementary file 2 — Additional file 2. GRIPP2 reporting checklist. [file 12911_2020_1022_MOESM2_ESM.pdf]

Supplementary S2: GRIPP2 reporting checklist

Section and topic	Item	Reported on page No
**1: Aim**	Report the aim of PPI in the study	6
**2: Methods**	Provide a clear description of the methods used for PPI in the study	7,8
**3: Study results**	Outcomes—Report the results of PPI in the study, including both positive and negative outcomes	12 - 14
**4: Discussion and conclusions**	Outcomes—Comment on the extent to which PPI influenced the study overall. Describe positive and negative effects	15
**5: Reflections/critical perspective**	Comment critically on the study, reflecting on the things that went well and those that did not, so others can learn from this experience	15
