# Supplementary material for: The development of the evidence-based SDMMCC intervention to improve shared decision making in geriatric outpatients: the DICO study
Source: BMC Med Inform Decis Mak. 2020 Feb 19;20:35. doi: 10.1186/s12911-020-1022-6 (PMC7031985; doi:10.1186/s12911-020-1022-6)
Supplement: Supplementary file 3 — Additional file 3. Heatmap Adaptions training SDM for geriatricians. [file 12911_2020_1022_MOESM3_ESM.pdf]

Supplementary S3 Heatmap Adaptions training SDM for geriatricians

The Dynamic model of SDM in frail old patients	Recommendations from Phase I	In SDM training general practitioners	Remove or add to SDM training geriatricians
1. Preparation			
1a. History: did the patients previously discuss anything with regard to treatment or other issues?	Emphasises investigating the patient's history		less attention in training
1b. Problem analysis: functional assessment of all current problems	Awareness of possible low health literacy or cognitive impairments & consequences for tailoring information		more attention in training
2. Goal talk			
2a. Explains to the patient that a new problem has occured and states that choices need to be made	Explicit invitation to participate in decision making		remains the same
2b. Explains that every patient is unique and has his own preferences and priorities	Addresses goals and expectations of patient, adress quality of life		more attention in training
2c. Engages the patient in a dialogue to clarify several important general topics that require clarification before choices can be made: Identifies discussion partner, addresses patient willingness to make shared decisions and identifies patient values and goals.	Discuss how informal caregiver should/wants to be involved		more attention in training
	Focuses on building a trust-relationship between clinician and patient		more attention in training
3. Choice talk			
3a. Summarizes the preceding steps and verifies if the recapitulation is correct	×		more attention in training
3b. Explains that there are several treatment possibilities and offers choice in different treatment possibilities	Attention for patient's preference to receive information		more attention in training
3c. Invites the atient to formulate their treatment aims and supports the patient: there are 2 expert in the medical encounter, checks if patient has understood everything and continues to engage patient in a dialogue.	Emphasize partnership geriatrician, patient, informal caregiver		more attention in training
4. Option talk			
4a. Lists personalised treatment options			remains the same
4b. Discusses risks, benefits and side effects of every option			remains the same
4c. Checks which risks and side effects the patient is willing to take			more attention in training
4d. Observes how the patient reacts and continues to engage the patient			more attention in training
4e. If possible uses decision aids			more attention in training
5. Decision talk			
5a. Inquires if the patient is ready to make a decision. If not, goes back to the preceding steps			much more attention in training
5b. Focuses on engaging a dialogue. Focuses on the preferences of a patient and makes a decision with the patient			remains the same
6. Evaluation			
6a. Dicusses the decision-making process. Is everybody satisfied?	Reflect on decision making proces, make treatment plan		much more attention in training

sufficiently addressed

moderately addressed

not/minimally adressed
